# Dementia-related continuing education for rural interprofessional primary health care in Saskatchewan, Canada: perceptions and needs of webinar participants

**DOI:** 10.1017/S1463423622000226

**Published:** 2022-05-23

**Authors:** Julie Kosteniuk, Debra Morgan, Megan E. O’Connell, Dallas Seitz, Valerie Elliot, Melanie Bayly, Chelsie Cameron, Amanda Froehlich Chow

**Affiliations:** 1 Canadian Centre for Health and Safety in Agriculture, College of Medicine, University of Saskatchewan, Saskatoon, Saskatchewan, Canada; 2 Department of Psychology, University of Saskatchewan, Saskatoon, Saskatchewan, Canada; 3 Department of Psychiatry, Cumming School of Medicine, University of Calgary, Calgary, Alberta, Canada; 4 School of Public Health, University of Saskatchewan, Saskatoon, Saskatchewan, Canada

**Keywords:** continuing education, dementia, primary health care, rural health

## Abstract

Dementia-related continuing education opportunities are important for rural primary health care (PHC) professionals given scarce specialized resources. This report explores the initial perceptions and continuing education needs of rural interprofessional memory clinic team members and other PHC professionals related to a short series of dementia-related education webinars. Three webinars on separate topics were delivered over an 8-month period in 2020 in Saskatchewan, Canada. The research design involved analysis of webinar comments and post-webinar survey data. Sixty-eight individuals participated in at least one webinar, and 46 surveys were completed. Rural memory clinic team members accounted for a minority of webinar participants and a majority of survey respondents. Initial perceptions were positive, with webinar topics and interactivity identified as the most effective aspects. Continuing education needs were mainly aligned with professional roles; however, some overlap of interests occurred. Future webinars will further explore learning needs within an interprofessional environment.

## Introduction

Interprofessional primary health care (PHC) is recognized as highly effective in terms of outcomes for patients with complex health needs such as dementia, as well as their care partners (Dreier-Wolfgramm *et al*., 2017; Samus *et al*., 2018; Heintz *et al*., 2020). While interprofessional PHC approaches vary in terms of professions involved and services provided, decision-making responsibility is commonly centred in the local PHC team most familiar with patients and available community resources, with support from dementia specialists through training and mentoring (Dreier-Wolfgramm *et al*., 2017; Heintz *et al*., 2020). In a recent review of interprofessional PHC teams providing dementia care to rural populations, Froehlich Chow *et al*. (2019) noted that ensuring team capacity through comprehensive training of all team members was essential.

Interprofessional education (IPE) involves two or more professions learning with, from, and about each other to improve collaboration and health outcomes (WHO, 2010). Meant to foster respect and trust between professions, IPE provides opportunities for group learning experiences to improve communication, teamwork, understanding of roles, and care coordination (WHO, 2010; Miller *et al*., 2019; Gonçalves *et al*., 2021; Huda, 2021). Theories informing IPE learning processes and activities represent constructivist (e.g. adult learning), intergroup process (e.g. contact hypothesis), and social constructionist perspectives (e.g. situated learning/communities of practice) (Barr, 2013; Hean *et al*., 2018). Common in IPE activities, principles of adult learning encourage cooperation between learners and reflection on one’s own views and those of others, drawing on group-based activities such as discussion, role play, and the use of real-life scenarios or clinical cases. For IPE activities to be effective, the contact hypothesis suggests several conditions beyond just contact between professions are necessary, for example ensuring participants have equal status and positive expectations of the activity (Carpenter and Dickinson, 2016). Situated learning or communities of practice regarding IPE activities refer to contexts characterized not by individual learning but by a sense of learning within a community developed through mutual engagement and shared resources (Barr *et al*., 2013; Hean *et al*., 2018).

IPE in primary care that promotes interaction and learning between different professional groups is important for strengthening collaboration skills and behaviours and fostering collaboration within teams (Donnelly *et al*., 2019; Miller *et al*., 2019). Studies indicate that interprofessional dementia training for primary care providers results in improved confidence and knowledge (Mastel-Smith *et al*., 2020), and participants appreciate opportunities to learn more about other professionals’ perspectives and practice roles (Jennings *et al*., 2019). Interprofessional continuing education opportunities may be especially meaningful in rural areas where specialized resources are rare, and other professionals in addition to family physicians are routinely responsible for recognizing and managing patients with dementia (Doyle *et al*., 2016; Bryan and Asghar-Ali, 2020).

Interprofessional training related to dementia in PHC is receiving increasing attention, with programmes ranging from short workshops for teams to multi-year undergraduate programmes (Dreir-Wolfgramm *et al*., 2017; Draper *et al*., 2018; Jennings *et al*., 2019; Mastel-Smith *et al*., 2020). In this study, we introduced a synchronous dementia-related continuing education webinar series for rural interprofessional PHC memory clinic teams as part of an ongoing research program. The research questions were as follows: how do webinar participants perceive the webinar content and format and what are the education needs of participants?

## Background

In 2017, the researchers collaborated with a PHC team in Saskatchewan, Canada to develop and implement the first rural interprofessional PHC memory clinic in the province (Morgan *et al*., 2019). One-day memory clinics are now offered every 1-2 months in four communities (population 300–11 000) by memory clinic teams consisting of PHC team members. The composition of each memory clinic team (hereafter team) varies, however included are either a family physician (FP) or nurse practitioner (NP) as lead clinician, home care nurse (HCN), allied health professionals (AHPs; social worker, occupational therapist, and physical therapist), and an Alzheimer Society First Link Coordinator.

Teams take part in training prior to establishing a memory clinic and ongoing mentoring thereafter, including continuing education sessions. Initial training is provided by Rural Dementia Action Research (RaDAR) team members and the developer (DS) of the Primary Care Dementia Assessment and Treatment Algorithm (PC-DATA™), which has been adapted and integrated into the clinic model. Prior to 2020, continuing education was offered periodically to one team at a time, delivered by clinical specialists and other experts via telehealth videoconference. Teams suggested topics in which they were keen to receive further training, which included differential diagnosis, capacity and competency, medication for dementia, and driving assessment. More teams were invited to take part in the sessions as the model spread to other communities. In a recent process evaluation, teams indicated participation in training and continuing education improved self-confidence in abilities and supported implementation of memory clinics (Morgan *et al*., 2019).

In February 2020, the researchers introduced a cross-team dementia-related continuing education series delivered via WebEx. Three webinars were delivered in 2020 on topics suggested by the teams and are included in this analysis (Supplemental Table 1). Each webinar began with a 30–45 minute presentation followed by 30–45 minutes of discussion. Team members had the flexibility to join remotely with their chosen device since they often travel between settings and communities. During the webinars, the presenter’s camera and audio were live and participants could offer written or oral comments at any time; however, participant’s cameras were turned off.

## Methods

The research design involved three post-webinar cross-sectional surveys and analysis of questions and comments offered by participants during the webinars. Four teams as well as NPs in PHC sites in nearby communities were invited to participate in webinars held in February, June, and September 2020. The third webinar on the topic of legal capacity was considered to have broad appeal outside memory clinic teams, and therefore, additional staff from the same geographic area were invited (e.g. long-term care employees).

Survey data were collected immediately after each webinar by Survey Monkey. Survey items included three demographic, nine Likert, and five open-ended questions (Supplemental Table 2). Six Likert items and two open-ended questions were adapted from previous evaluations of dementia-related continuing education sessions for health professionals (Doyle *et al*., 2016; Bryan and Asghar-Ali, 2020; Yous *et al*., 2020); the remaining items were developed by the research team. Because all memory clinic team members with the exception of one individual were female, survey respondent sex was not requested. The webinars were recorded and transcribed, and survey data were exported to Excel (2021). Qualitative data from webinar questions/comments and open-ended survey items were analysed descriptively to identify themes, and descriptive statistics (frequencies and proportions) were used to analyse quantitative survey data.

## Results

Sixty-eight individuals participated in at least one webinar, and 46 post-webinar surveys were completed. Across all webinars, most participants belonged to the nursing profession (Table 1). A minority of webinar participants were memory clinic team members; however, this group comprised the majority of survey respondents. The survey response rate ranged from 42% to 67% across the three webinars, with FP/NPs accounting for the majority of survey respondents (Table 2).

**Table 1.** Characteristics of webinar participants

	All webinars (*N* = 68)^a^ *n* (%)	Webinar 1 Medication and substance-induced cognitive impairment (*N* = 24) *n* (%)	Webinar 2 Management of behavioural symptoms of dementia in long-term care (*N* = 21) *n* (%)	Webinar 3 Legal capacity (*N* = 48) *n* (%)
Memory clinic team member				
Yes	21 (30.9)	16 (66.7)	15 (71.4)	10 (20.8)
No	47 (69.1)	8 (33.3)	6 (28.6)	38 (79.2)
Professional role				
Family physician	3 (4.4)	3 (12.5)	1 (4.8)	0 (0)
Nurse practitioner/registered nurse/Licensed practical nurse	27 (39.7)	12 (50.0)	9 (42.9)	16 (33.3)
Allied health professional	14 (20.6)	6 (25.0)	8 (38.1)	11 (22.9)
Other professional^b^	19 (27.9)	3 (12.5)	3 (14.3)	16 (33.3)
Missing	5 (7.4)	0	0	5 (10.4)

a 19/68 participants attended multiple webinars.

b Administrator (executive, senior leader, manager, director), Primary Health Care Facilitator, Alzheimer Society staff, and other roles.

**Table 2.** Characteristics of survey respondents

	All webinars (*N* = 46) *n* (%)	Webinar 1 Medication and substance-induced cognitive impairment (*N* = 16) *n* (%)	Webinar 2 Management of behavioural symptoms of Dementia in Long-term Care (*N* = 10) *n* (%)	Webinar 3 Legal capacity (*N* = 20) *n* (%)
Memory clinic team member				
Yes	29 (64.0)	12 (75.0)	6 (60.0)	11 (55.0)
No	16 (34.8)	3 (18.8)	4 (40.0)	9 (45.0)
Missing	1 (2.2)	1 (6.3)	0	0
Professional role				
Family physician/nurse practitioner	14 (30.4)	7 (43.8)	6 (60.0)	1 (5.0)
Home care nurse	7 (15.2)	2 (12.5)	0	5 (25.0)
Allied health professional	13 (28.2)	4 (25.0)	2 (20.0)	7 (35.0)
Other professional^a^	11 (23.9)	2 (12.5)	2 (20.0)	7 (35.0)
Missing	1 (2.2)	1 (6.3)	0	0
Age				
Under 30	6 (13.0)	3 (18.8)	2 (20.0)	1 (5.0)
30–39	16 (34.8)	5 (31.3)	3 (30.0)	8 (40.0)
40–49	13 (28.3)	5 (31.3)	3 (30.0)	5 (25.0)
50–59	10 (21.7)	2 (12.5)	2 (20.0)	6 (30.0)
Missing	1 (2.2)	1 (6.3)	0	0

a Administrator (executive, senior leader, manager, director), Primary Health Care Facilitator, and Alzheimer Society staff.

Among survey respondents across the webinars, overall satisfaction was high (94%) (Supplemental Table 3). Regarding webinar content, the majority agreed the sessions were appropriate for their professional needs and new information was learned (96%). Most found the interactive format and webinar environment effective for learning (96%). A majority also intended to apply the information in their practice and appreciated the participation of other PHC teams and professionals; however, these particular items were endorsed by fewer participants (<90%).

In open-ended comments, the most effective aspects of the webinar identified by survey respondents were the webinar topics and interactive question and answer format (Table 3). Primarily AHPs commented on topic effectiveness (*n* = 6 of 10 comments) (data not shown in table). Regarding the first two webinars which were more medication-focused than the third session, some AHPs found themselves engaged although they were not in a prescribing role and did not feel highly knowledgeable about the topic (Table 3 quotations). Interactivity effectiveness was cited by most groups (FP, AHP, and HCN), particularly the benefit of hearing others’ questions which resulted in ‘deeper understanding’. Other effective aspects included clarity of the presentations, use of case studies, and presenters’ knowledge. The three least effective aspects were ‘nothing’ (everything was considered effective), medication focus, and limited time, with AHPs putting forward all comments regarding medication concentration. Contrary to the positive feedback of some AHPs toward the medication-focused webinars, other AHPs noted they felt ‘lost in the knowledge’, had little use for such information because they were not involved in prescribing, and found information about specific medications to be ineffective.

**Table 3.** Themes identified in survey responses: Most and least effective aspects of webinars

	Representative quotation	All webinars (*N* = 46) *n*	Webinar 1 Medication and substance-induced cognitive impairment (*N* = 16) *n*	Webinar 2 Management of behavioural symptoms of dementia in long-term care (*N* = 10) *n*	Webinar 3 Legal capacity (*N* = 20) *n*
Most effective aspect					
Topic	‘I don’t prescribe but found the presentation very interesting’. (Allied Health Professional, Webinar 1)	10	2	3	5
Interactive question and answer (Q and A)	‘The Q&A is always good to have. Some ask questions that I had not considered. Deeper understanding gained for same’. (Home Care Nurse, Webinar 3)	6	4	0	2
Presentation clarity	‘The information was explained well and yet understandable to those of us that are not as knowledgeable about medications’. (Allied Health Professional, Webinar 2)	5	0	3	2
Case presentations and examples	‘I really enjoy listening to [the presenter] and hearing [their] perspective on the case studies…’ (Family Physician/Nurse Practitioner, Webinar 1)	5	3	0	2
Presenter	‘[The presenter’s] knowledge on the topic was amazing’. (Other Professional^a^, Webinar 3)	5	1	2	2
Clinical applicability	‘The presenter […] provided excellent information I will apply to my practice’. (Family Physician/Nurse Practitioner, Webinar 2)	3	0	2	1
Least effective aspect					
Nothing (everything was effective)	‘Nothing seemed to be unnecessary!’ (Family Physician/Nurse Practitioner, Webinar 1)	7	3	1	3
Medication focus	‘I have no involvement in the prescribing of medications so it was interesting but not something I can use in my practice’. (Allied Health Professional, Webinar 2)	3	1	2	0
Limited time	‘I thought it could be a little longer in length as the presenter went over the material and answered the questions quite quickly’. (Home Care Nurse, Webinar 3)	3	0	0	3
Presenter	‘[The presenter] didn’t seem sure of some of [their] answers but I understand [the topic] isn’t always clear’. (Allied Health Professional, Webinar 3)	2	0	0	2

a Administrator (executive, senior leader, manager, director), Primary Health Care Facilitator, or Alzheimer Society staff.

Asked to suggest future webinar topics, FP/NP survey respondents recommended education on preventing decline, avoiding polypharmacy, and insomnia (Table 4). AHPs further recommended topics related to dementia type, caregiver resources, and nutrition. Both FP/NP and AHP groups were interested in management using pharmacological and non-pharmacological interventions, and legal capacity topics were of interest to AHP and HCN groups.

**Table 4.** Survey respondents’ suggestions for future webinar topics and representative quotations

Topic (*n*)	Representative quotation
Family physician/nurse practitioner	
Resources to prevent decline (1)	‘Also looking at different resources we can implement to help/prevent decline in our patients with dementia […]’ (Webinar 1)
Avoiding polypharmacy (2)	‘Withdrawal of therapies in people with dementia, ie how to avoid polypharmacy. When do we withdraw other meds, diabetes, cholesterol, etc.’ (Webinar 2)
Insomnia (1)	‘Insomnia’ (Webinar 2)
Allied health professional	
Pathophysiology of different types of dementia (1)	‘Pathophysiology of various types of dementias (i.e. what areas/tracts of the brain are affected in each and how that manifests in physical and/or cognitive symptoms specific to that condition)’. (Webinar 1)
Nutrition therapy (1)	‘Nutrition therapy in dementia’. (Webinar 1)
Caregiver support and home-based resources (1)	‘Support for caregivers, knowledge of tools and resources for remaining in home’. (Webinar 2)
Assessing competency and capacity [legal capacity] (2^a^)	‘I would like to learn more about capacity itself and deeming someone incompetent and such. The process, who is involved, what is needed’. (Webinar 3)
Family physician/nurse practitioner and allied health professional	
Pharmacological and non-pharmacological management (4)	‘[…] different treatments/interventions we can use both pharmacological, and non-pharmacological interventions. With our [anonymized] [memory] clinics they are great at helping us make that diagnosis and setting them up to succeed at home, but then what?’ (Family Physician/Nurse Practitioner, Webinar 1) ‘Medication effects/usefulness on functional performance (i.e. Aricept and gait/balance in AD versus VD versus LBD)’. (Allied Health Professional, Webinar 1)
Any topic (2)	‘Whatever [the presenter] wants to talk about’ (Family Physician/Nurse Practitioner, Webinar 1)
Allied health professional and home care nurse	
Proxy and guardianship [legal capacity] (3)	‘I would like to see information presented in relation to proxy and guardianship in regards to health care needs’. (Home Care Nurse, Webinar 3)

a Suggested by one Allied Health and one Other Professional [Administrator (executive, senior leader, manager, director), Primary Health Care Facilitator, or Alzheimer Society staff].

During the first two webinars, participant questions/comments were volunteered mainly by NPs and centred on medication management, pain assessment and control, and depression treatment, in line with the actual webinar topics. All questions during the third webinar were related to the presentation topic of legal capacity and were put forward by members of all professional groups in attendance (NP/Registered Nurse/Licensed Practical Nurse, AHP, and Other). Illustrative quotations of themes are provided in Supplemental Table 4.

## Discussion

This study explored the perceptions and continuing education needs of rural memory clinic team members and other PHC professionals participating in interprofessional dementia-focused webinars. Findings from post-webinar survey evaluations indicated positive initial perceptions of webinar content and format, and education suggestions that mainly corresponded to professional roles.

Our findings are consistent with research showing rural health professionals react positively to dementia care learning opportunities delivered remotely (Doyle *et al*., 2016). Research suggests continuing education by distance is equivalent to in-person learning with respect to knowledge and satisfaction outcomes of rural health care providers (Berndt *et al*., 2017). Distance education allows urban-based dementia specialists, often in short supply, to support isolated health professionals (Doyle *et al*., 2016) and may result in impact beyond the original learners if information is disseminated to rural colleagues (Sass *et al*., 2019). Prior to the COVID-19 pandemic, accessing urban-based training from rural locations required technical infrastructure and expertise that may now be more commonplace. In this study, acceptance of the webinar format was possibly facilitated by increased comfort with virtual meetings fostered during the pandemic (Prasad *et al*., 2020).

Survey respondents identified session topics as the most effective webinar feature, possibly due partly to our efforts to organize webinars that met teams’ earlier topic suggestions. Tailoring dementia care education to the needs of interprofessional PHC teams contributes to the success of such initiatives (Bryan and Asghar-Ali, 2020). Some members of the AHP group demonstrated mixed perceptions regarding the effectiveness of the medication focus of the first two webinars, citing low involvement in prescribing. Similarly, previous research notes varying levels of dementia care experience and training among learners can pose a challenge when offering IPE (Dreier-Wolfgramm *et al*., 2017). However, satisfaction with webinar content among survey participants was high suggesting interprofessional learning may be enhanced by conveying technical information in a clear and organized fashion, using case studies with wide appeal, and using an interactive format that encourages participation. In this study, interactivity was cited as effective consistent with findings of previous research regarding the importance of active learner engagement (Berndt *et al*., 2017), particularly in web-based dementia learning formats (Scerbe *et al*., 2019). Compared to didactic approaches that rely on more passive learning, a review of continuing education for health workers found teaching techniques that incorporate interaction and encourage critical thinking (e.g. case studies and simulation) produce better knowledge outcomes (Bluestone *et al*., 2013). Interactivity during interprofessional training allows learners the benefit of hearing questions/comments from different professional perspectives, possibly supporting learning about both the topic and scope of others’ professional practice. Interactivity between rural AHP learners taking part in continuing education has been shown to also facilitate networking (Berndt *et al*., 2017). Whether an interactive learning approach complements and strengthens relationships and activities within existing interprofessional teams are topics for future research.

Suggestions by survey respondents for future education topics generally aligned with professional roles. The mutual interest of AHPs and FP/NPs in pharmacological and non-pharmacological management, and emphasis by FP/NPs on management interventions, underscores the challenge of these issues in primary care. Interest in medication-related topics is consistent with recent research showing potentially inappropriate prescribing is associated with higher levels of comorbidity among people living with dementia (Delgado *et al*., 2020). Fewer than half of Canadian primary care doctors report feeling well prepared to manage care for patients with dementia (Canadian Institute for Health Information, 2018), and a recent study found feelings related to helplessness in dementia management more prevalent among family physicians who lacked dementia-related training (Heim *et al*., 2019). To help improve management capacity, the rural memory clinic model and other task-sharing approaches collaborate with highly qualified and experienced dementia specialists to further train and support PHC professionals (Canadian Academy of Health Sciences, 2019; Heintz *et al*., 2020), whereas deprescribing interventions focus primarily on physician prescribing behaviour (Parsons, 2017), the educational needs of other PHC professionals in the circle of care and of NPs should be considered as well, given NPs broad scope of practice in rural settings that often includes independent medication prescribing (Macleod *et al*., 2017). Consistent with previous research (Lee *et al*., 2020), education interests among interprofessional memory clinic members in this study diverged and overlapped, indicating that some may seek to broaden their knowledge beyond traditional roles, particularly in rural communities with fewer dementia-specific resources. Future research could examine the knowledge and skill levels of webinar participants along a novice-expert continuum (e.g. managing patients with complex needs). Longer follow-up would be necessary to examine whether professional practice improved. Further exploration of dementia-related education needs of rural interprofessional providers and strategies to meet these needs are potential directions for further research.

Limitations of this study should be considered. Survey items focused only on a few selected perceptions of webinar content and format. Other measures of content and format, and other domains, may have been pertinent to participant experience but were not included. Survey participation was voluntary; therefore, the results are subject to non-response bias. Respondents may also have tended to agree rather than disagree with statements, reflecting acquiescence bias. Furthermore, the initial findings from a small sample of professionals from one geographic area limit the generalizability of the findings.

## Conclusion

There have been few published studies of initiatives that offer dementia-related continuing education in an interprofessional setting to PHC professionals. Initial results from this study indicate favourable feedback from rural participants of a short series of interprofessional dementia-related education webinars. The findings also revealed opportunities to seek further input on varying education needs within teams, to inform future webinars and research.

## Supplementary material

For supplementary material accompanying this paper visit http://doi.org/10.1017/S1463423622000226. click here to view supplementary material

## References

[ref2] Barr H (2013) Toward a theoretical framework for interprofessional education. Journal of Interprofessional Care 27, 4–9. 22747337 10.3109/13561820.2012.698328

[ref3] Berndt A , Murray C , Kennedy K , Stanley M and Gilbert-Hunt S (2017) Effectiveness of distance learning strategies for continuing professional development (CPD) for rural allied health practitioners: a systematic review. BMC Medical Education 17, 117. 28701199 10.1186/s12909-017-0949-5 PMC5506644

[ref4] Bluestone J , Johnson P , Fullerton J , Carr C , Alderman J and BonTempo J (2013) Effective in-service training design and delivery: evidence from an integrative literature review. Human Resources for Health 11, 51. 24083659 10.1186/1478-4491-11-51 PMC3850724

[ref5] Bryan J and Asghar-Ali A (2020) Development and dissemination of an interprofessional online dementia training curriculum. Journal of the American Geriatrics Society 68, 192–197. 31693177 10.1111/jgs.16240

[ref6] Canadian Academy of Health Sciences (2019) Improving the quality of life and care of persons living with dementia and their caregivers. Ottawa (ON): The Expert Panel on Dementia Care in Canada, CAHS.

[ref8] Canadian Institute for Health Information (2018) Dementia in Canada. Ottawa, ON: CIHI.

[ref9] Carpenter J and Dickinson C (2016) Understanding interprofessional education as an intergroup encounter: the use of contact theory in programme planning. Journal of Interprofessional Care 30, 103–108. 26833110 10.3109/13561820.2015.1070134

[ref90] Delgado J , Bowman K and Clare L (2020) Potentially inappropriate prescribing in dementia: a state-of-the-art review since 2007. BMJ Open 10, e029172. 10.1136/bmjopen-2019-029172 PMC6955517 31900263

[ref10] Donnelly C , Ashcroft R , Mofina A , Bobbette N and Mulder C (2019) Measuring the performance of interprofessional primary health care teams: understanding the teams perspective. Primary Health Care Research and Development 20, 1–8. 10.1017/S1463423619000409 PMC6719251 31455458

[ref11] Doyle C , Jackson D , Loi S , Malta S and Moore K (2016) Videoconferencing and telementoring about dementia care: evaluation of a pilot model for sharing scarce old age psychiatry resources. International Psychogeriatrics 28, 1567–1574. 27189501 10.1017/S1041610216000740

[ref12] Draper B , Low LF and Brodaty H (2018) Integrated care for adults with dementia and other cognitive disorders. International Review of Psychiatry 30, 272–291. 30810424 10.1080/09540261.2018.1564021

[ref13] Dreier-Wolfgramm A , Michalowsky B , Austrom M , van der Marck M , Iliffe S , Alder C , Vollmar H , Thhrian J , Wucherer D , Zwingmann I and Hoffman W (2017) Dementia care management in primary care. Journal for Gerontology and Geriatrics 50, S68–S67. 10.1007/s00391-017-1220-8 28364258

[ref14] Froehlich Chow A , Morgan D , Bayly M , Kosteniuk J and Elliot V (2019) Collaborative approaches to team-based primary health care for individuals with dementia in rural/remote settings. Canadian Journal on Aging 38, 367–383. 30846013 10.1017/S0714980818000727

[ref15] Gonçalves J , Gonçalves R , Rosa S , Orsi J , Moysés J and Werneck R (2021) Impact of interprofessional education on the teaching and learning of higher education students: a systematic review. Nurse Education in Practice 56, 103212. 34571466 10.1016/j.nepr.2021.103212

[ref16] Hean S , Green C , Anderson E , Morris D , John C , Pitt R and O’Halloran C (2018) The contribution of theory to the design, delivery, and evaluation of interprofessional curricula: BEME Guide No. 49. Medical Teacher 40, 542–558. 29457926 10.1080/0142159X.2018.1432851

[ref17] Heim S , Busa C , Pozsgai E , Csikós A , Papp E , Pákáski M , Kálmán J , Hajnal F and Karádi K Hungarian general practitioners’ attitude and the role of education in dementia care. Primary Health Care Research & Development 20, 1–6. 10.1017/S1463423619000203 PMC6609992 32799975

[ref18] Heintz H , Monette P , Epstein-Lubow G , Smith L , Rowlett S and Forester B (2020) Emerging collaborative care models for dementia care in the primary care setting: a narrative review. American Journal of Geriatric Psychiatry 3, 320–330. 10.1016/j.jagp.2019.07.015 31466897

[ref19] Huda N (2021) Building the primary healthcare workforce for interprofessional collaboration. Liaquat National Journal of Primary Care 3, 56–57.

[ref20] Jennings A , McLoughlin K , Boyle S , Thackeray K , Quinn A , O’Sullivan T and Foley T (2019) Development and evaluation of a primary care interprofessional education intervention to support people with dementia. Journal of Interprofessional Care 33, 579–582. 30422731 10.1080/13561820.2018.1541876

[ref21] Lee L , Hillier L , Patel T and Weston W (2020) A decade of dementia care training: learning needs of primary care physicians. Journal of Continuing Education in the Health Professions 40, 131–140. 32175933 10.1097/CEH.0000000000000288

[ref22] MacLeod M , Stewart N , Kulig J , Jonatansdottir S , Olynick J and Kosteniuk J (2017) *Nurse Practitioner National Survey Fact Sheet: Nursing Practice In Rural and Remote Canada.* Nursing Practice in Rural and Remote Canada II. https://www2.unbc.ca/rural-nursing/en/publications.

[ref23] Mastel-Smith B , Kimzey M , Garner J , Shoair O , Stocks E and Wallace T (2020) Dementia care boot camp: interprofessional education for healthcare students. Journal of Interprofessional Care 34, 799–811. 31835917 10.1080/13561820.2019.1696287

[ref24] Miller R , Scherpbier N , Amsterdam L , Guedes V and Pype P (2019) Inter-professional education and primary care: EFPC position paper. Primary Health Care Research and Development 20, 1–10. 10.1017/S1463423619000653 PMC6784359 31581968

[ref1] Morgan D , Kosteniuk J , O’Connell ME , Kirk A , Stewart N , Seitz D , Bayly M , Froehlich Chow A , Elliot V , Daku J , Hack T , Hoium F , Kennett-Russill D and Sauter K (2019) Barriers and facilitators to development and implementation of a rural primary health care intervention for dementia: A process evaluation. BMC Health Services Research 19, 709. 31623609 10.1186/s12913-019-4548-5 PMC6798332

[ref25] Parsons C (2017) Polypharmacy and inappropriate medication use in patients with dementia: an under-researched problem. Therapeutic Advances in Drug Safety 8, 31–46. 28203365 10.1177/2042098616670798 PMC5298466

[ref26] Prasad N , Fernando S , Willey S , Davey K , Kent F , Malhotra M and Kumar A (2020) Online interprofessional simulation for undergraduate health professional students during the COVID-19 pandemic. Journal of Interprofessional Care 34, 706–710. 32917099 10.1080/13561820.2020.1811213

[ref28] Samus Q , Black B , Bovenkamp D , Buckley M , Callahan C , Davis K , Gitlin L , Hodgson N , Johnston D , Kales H , Karel M , Kenney J , Ling S , Panchal M , Reuland M , Willink A and Lyketsos C (2018) Home is where the future is: the BrightFocus Foundation consensus panel on dementia care. Alzheimers & Dementia 14, 104–114. 10.1016/j.jalz.2017.10.006 PMC5870894 29161539

[ref27] Sass C , Burnley N , Drury M , Oyebode J and Surr C (2019) Factors associated with successful dementia education for practitioners in primary care: an in-depth case study. BMC Medical Education 19, 393. 31660920 10.1186/s12909-019-1833-2 PMC6819571

[ref29] Scerbe A , O’Connell M , Astell A , Morgan D , Kosteniuk J and DesRoches A (2019) Digital tools for delivery of dementia education for health-care providers: a systematic review. Educational Gerontology 25, 681–699.

[ref30] World Health Organization (WHO) (2010) Framework for action on interprofessional education and collaborative practice. Geneva: WHO. 21174039

[ref31] Yous M-L , Ploeg J , Kaasalainen S and Schindel Martin L (2020) Healthcare professionals’ perceptions of PIECES education in supporting care delivery for older adults with responsive behaviours of dementia in acute care. Gerontology & Geriatrics Education 41, 32–51. 30704367 10.1080/02701960.2019.1572011

