# Supplementary material for: Dementia-related continuing education for rural interprofessional primary health care in Saskatchewan, Canada: perceptions and needs of webinar participants
Source: Prim Health Care Res Dev. 2022 May 23;23:e32. doi: 10.1017/S1463423622000226 (PMC9247685; doi:10.1017/S1463423622000226)
Supplement: Supplementary file 1 [file S1463423622000226sup001.docx]

Supplemental Table 1. Webinar presenters, topics, and objectives

|  | Webinar 1  Medication and Substance-induced Cognitive Impairment | Webinar 2  Management of Behavioural Symptoms of Dementia in Long-term Care | | | Webinar 3  Legal Capacity | | |  |
| --- | --- | --- | --- | --- | --- | --- | --- | --- |
| Speaker | Geriatric Psychiatrist | Geriatric Psychiatrist | | Senior Crown Counsel | | | | |
|  |  |  | |  | | | | |
| Duration | 90 minutes | 90 minutes | | 75 minutes | | | | |
|  |  |  | |  | | | | |
| Topics and objectives | - Review some presentations of cognitive impairment that may be caused by medications or other substances - Understand common medications or substances that can contribute to cognitive impairment - Develop an approach to managing individuals with substance induced cognitive impairment | | - Understand factors that contribute to the development of neuropsychiatric symptoms (NPS) - Review recent developments in non-pharmacological and pharmacological treatments for NPS - Apply this knowledge in clinical settings | | | - Review the role of the Saskatchewan Public Guardian and Trustees Office - Review standards and assessment process of legal capacity for wills, powers of attorney, healthcare directives, and certificates and forms of incapacity |  |  |

Supplemental Table 2. Webinar and survey items

| Webinar items | Survey items |
| --- | --- |
| Open-ended questions and comments volunteered by participants  Professional role (8 categories)  Memory clinic team member (yes/no) | Age (5 categories)  Professional role (8 categories)  Memory clinic team member (yes/no)  Likert scale items (strongly agree, agree, neutral, disagree, strongly disagree)   - Webinar content (4 items) - Webinar format (3 items) - Overall satisfaction (2 items)   Open-ended questions   - Most effective aspect of webinar - Least effective aspect of webinar - Future webinar topic suggestions - Most useful aspect of session structure^a^ - Other comments^a^ |

^a^ Not included in analysis

Supplemental Table 3. Perceptions of survey respondents: Webinar effectiveness

|  | All webinars  (*N*=46)  *n* (%) | | | Webinar 1  Medication and Substance-induced Cognitive Impairment  (*N*=16)  *n* (%) | | | Webinar 2  Management of Behavioural Symptoms of Dementia in  Long-term Care  (*N*=10)  *n* (%) | | | Webinar 3  Legal Capacity  (*N*=20)  *n* (%) | | |
| --- | --- | --- | --- | --- | --- | --- | --- | --- | --- | --- | --- | --- |
|  | Agree | Neutral | Disagree | Agree | Neutral | Disagree | Agree | Neutral | Disagree | Agree | Neutral | Disagree |
| This session was appropriate for my professional needs | 44 (95.7) | 2 (4.3) | 0 | 15 (93.8) | 1 (6.3) | 0 | 9 (90.0) | 1 (10.0) | 0 | 20 (100) | 0 | 0 |
| This session met my expectations | 42 (91.3) | 4 (8.7) | 0 | 14 (87.5) | 2 (12.5) | 0 | 10 (100) | 0 | 0 | 18 (90.0) | 2 (10.0) | 0 |
| I learned new information during this session | 44 (95.7) | 2 (4.3) | 0 | 15 (93.8) | 1 (6.3) | 0 | 9 (90.0) | 1 (10.0) | 0 | 20 (100) | 0 | 0 |
| I intend to apply information from this session to my practice | 41 (89.1) | 5 (10.9) | 0 | 13 (81.3) | 3 (18.8) | 0 | 9 (90.0) | 1 (10.0) | 0 | 19 (95.0) | 1 (5.0) | 0 |
|  |  |  |  |  |  |  |  |  |  |  |  |  |
| The structure of this session was effective for my learning (i.e., presentation of a specific topic followed by open Q + A) | 44 (95.7) | 2 (4.3) | 0 | 16 (100) | 0 | 0 | 10 (100) | 0 | 0 | 18 (90.0) | 2 (10.0) | 0 |
| The WebEx environment of this session was effective for my learning | 44 (95.7) | 2 (4.3) | 0 | 15 (93.8) | 1 (6.3) | 0 | 10 (100) | 0 | 0 | 19 (95.0) | 1 (5.0) | 0 |
| The participation of members of other primary health care teams was effective for my learning^a,b^ | 40 (87.0) | 5 (10.9) | 0 | 15 (93.8) | 1 (6.3) | 0 | 7 (70.0) | 2 (20.0) | 0 | 18 (90.0) | 2 (10.0) | 0 |
|  |  |  |  |  |  |  |  |  |  |  |  |  |
| I would like to attend another education session offered by this presenter in the future | 44 (95.7) | 2 (4.3) | 0 | 16 (100) | 0 | 0 | 10 (100) | 0 | 0 | 18 (90.0) | 2 (10.0) | 0 |
|  |  |  |  |  |  |  |  |  |  |  |  |  |
| Overall, I was satisfied with this education session^b^ | 43 (93.5) | 2 (4.3) | 0 | 14 (87.5) | 2 (12.5) | 0 | 10 (100) | 0 | 0 | 19 (95.0) | 0 | 0 |

^a^ In the Webinar 3 survey, ‘members of other primary health care teams’ was revised to ‘other primary health care professionals’

^b^ 1 missing response

*Note.* Agree = Strongly Agree/Agree; Disagree = Strongly Disagree/Disagree

Supplemental Table 4. Webinar questions/comments, and representative quotations

|  |  |  | Representative quotation |  |
| --- | --- | --- | --- | --- |
| Question/Comment (*n*) | Webinar 1  Medication and Substance-induced Cognitive Impairment | | Webinar 2  Management of Behavioural Symptoms of Dementia in  Long-term Care | Webinar 3  Legal Capacity |
| Medication management (3) | “One of our last patients at our memory clinic, she had been on Aricept, and she had actually stopped it herself unbeknownst to us. And she really actually deteriorated quite quickly. Now, I put her back on it, just because she’d been doing so good. Now, is that the right thing to do or should I have just left that alone?” (Nurse Practitioner) | |  |  |
| Pain assessment and control (2) | “One of my patients that’s been to our memory clinics, she has lots of degenerated discs in her spine. And she’s having horrible pain. And she’s probably moderate to severe dementia at this point. And so the daughter tried using some CBD oil. It didn’t help her pain, I don’t think it really changed anything else. But I’m having a hard time trying to figure out how to control her pain without making her more confused. I don’t know what your thoughts are on that.” (Nurse Practitioner) | | “What are some of the tools that you use to assess symptoms like pain and behaviours?” (Nurse Practitioner) |  |
| Depression treatment (1) |  | | “What do you recommend [as] first line to treat depression in dementia?” (Nurse Practitioner) |  |
|  |  | |  |  |
| Capacity/competence assessment (2) |  | |  | “If someone is deemed financially incompetent (PGT paperwork is done) does that mean they are incompetent to make personal or healthcare decisions?” (Allied Health Professional) |
| Public Guardian and Trustee role and process (4) |  | |  | “How long does the process take to assign a trustee or guardian? Often these decisions are needed in a crisis situation, i.e. person unable to live alone and requires long term care.” (Allied Health Professional) |
| Power of attorney (2) |  | |  | “What’s the difference between power of attorney and personal guardianship?” (Nurse Practitioner) |

*Note*. Webinar 1 and 2 participants had the option to comment in verbal and written format; webinar 3 participants were able to comment in written format only.
